# Protease-Resistant Peptides for Targeting and Intracellular Delivery of Therapeutics

**DOI:** 10.3390/pharmaceutics13122065

**Published:** 2021-12-02

**Authors:** Maria C. Lucana, Yolanda Arruga, Emilia Petrachi, Albert Roig, Roberta Lucchi, Benjamí Oller-Salvia

**Affiliations:** Grup d’Enginyeria de Materials (GEMAT), Institut Químic de Sarrià (IQS), Universitat Ramon Llull, 08017 Barcelona, Spain; marialucanam@iqs.url.edu (M.C.L.); yolandaarrugat@iqs.url.edu (Y.A.); emilia.petrachi@iqs.url.edu (E.P.); albertroigm@iqs.url.edu (A.R.); roberta.lucchi@iqs.url.edu (R.L.)

**Keywords:** targeting peptides, cell-penetrating peptides, protease resistance, proteolysis, enantio, retro-enantio, retro-inverso, cyclic peptides

## Abstract

Peptides show high promise in the targeting and intracellular delivery of next-generation bio- and nano-therapeutics. However, the proteolytic susceptibility of peptides is one of the major limitations of their activity in biological environments. Numerous strategies have been devised to chemically enhance the resistance of peptides to proteolysis, ranging from *N*- and *C*-termini protection to cyclization, and including backbone modification, incorporation of amino acids with non-canonical side chains and conjugation. Since conjugation of nanocarriers or other cargoes to peptides for targeting and cell penetration may already provide some degree of shielding, the question arises about the relevance of using protease-resistant sequences for these applications. Aiming to answer this question, here we provide a critical review on protease-resistant targeting peptides and cell-penetrating peptides (CPPs). Two main approaches have been used on these classes of peptides: enantio/retro-enantio isomerization and cyclization. On one hand, enantio/retro-enantio isomerization has been shown to provide a clear enhancement in peptide efficiency with respect to parent L-amino acid peptides, especially when applied to peptides for drug delivery to the brain. On the other hand, cyclization also clearly increases peptide transport capacity, although contribution from enhanced protease resistance or affinity is often not dissected. Overall, we conclude that although conjugation often offers some degree of protection to proteolysis in targeting peptides and CPPs, modification of peptide sequences to further enhance protease resistance can greatly increase homing and transport efficiency.

## 1. Introduction

Targeted therapeutics are changing the established medical paradigm. This type of therapeutics is capable of selectively affecting desired cells, thereby dramatically reducing side effects. Although monoclonal antibodies have been the basis for the most successful targeted therapies until now, several alternatives are arising. In order to increase tissue selectivity of nano- and biotherapeutics other than antibodies, many efforts are devoted to exploring more accessible targeting moieties such as small molecules and peptides. Peptides, which are defined by the FDA as polymers composed of 40 or fewer amino acids [1], lie between small molecules and biotherapeutics and can combine the best of the two worlds. Similar to antibodies, peptides may possess a high affinity and selectivity, and, like small molecules, they are synthetically accessible, easy to derivatize, and generally have low immunogenicity. Moreover, peptides with particular physicochemical properties may help nano- and biotherapeutics cross the cell membrane, which is the ultimate barrier to achieving intracellular activity.

The main obstacles towards widespread applications of peptides are their rapid renal clearance and their susceptibility to proteolytic degradation. While the former is a concern for stand-alone peptide therapeutics, it is not a major problem when conjugated to protein therapeutics or nanomedicines. Conversely, proteolysis presents a potential problem to be addressed in any kind of peptide, either conjugated or not. The cause of the rapid degradation of peptides is the presence of endogenous proteases, which are enzymes that catalyze the hydrolysis of peptide bonds. Exoproteases attack terminal amino acids, while endoproteases may recognize motifs inside the peptide sequences and hydrolyze internal peptide bonds [2]. As a consequence, most linear peptides with all-L amino acids have a half-life of 5–30 min in serum [3], which would appear insufficient to enable efficient targeting, especially of large therapeutics.

Since many all-L linear peptides have successfully been used to target cargoes to several tissues [4], one might expect that the cargo molecule would offer some degree of protection against proteolysis due to steric hindrance. Despite the extensive literature describing peptides used as targeting and cell-penetrating moieties, little information is available about how peptide conjugation protects them from protease degradation. Hence, the question arises about the extent to which increasing the metabolic resistance of targeting peptides is relevant in conjugated peptides. Aiming to answer this question, we critically reviewed the recent literature and selected relevant examples to discuss the influence of peptide resistance to proteases in their targeting or cell penetration capacity. Herein, we first summarize the main strategies for increasing peptide resistance to proteases. Then, we focus on the two main strategies used to enhance stability in targeting and cell-penetrating peptides. Throughout the review, we illustrate with many examples how making peptides protease-resistant further boosts their targeting and transport efficiency.

## 2. Peptide Resistance to Proteases May Be Enhanced through a Variety of Chemical Modifications

**Figure 1:**
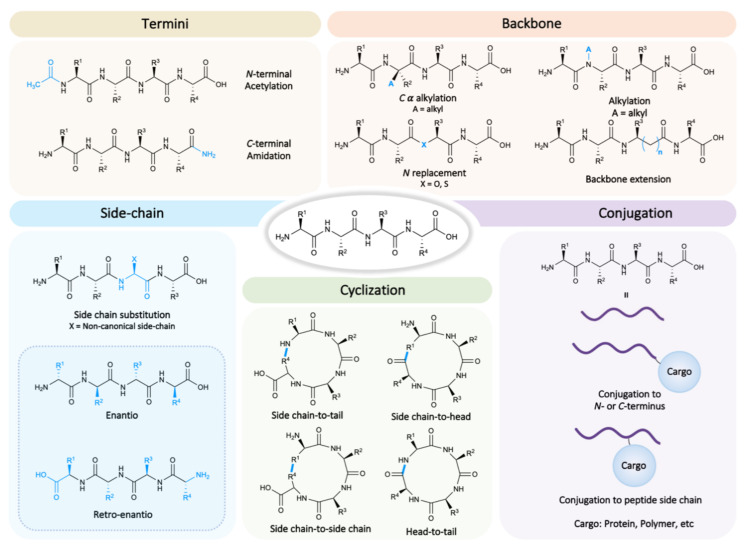
A plethora of strategies for enhancing peptide resistance to proteases have been developed.

Strategies for enhancing the resistance of peptides to proteases range from protection of *N*- and *C*-termini to conjugation, including backbone modification, incorporation of alpha amino acids with non-canonical side chains, and cyclization [5] (Figure 1). A combination of more than one strategy is also frequently used [6]. Peptides with such modifications can be included within the broad classification of “peptidomimetics” [7].

### 2.1. N- and C-Termini

The most basic form of protection is to modify the *N*- and *C*-termini of the peptide, thereby reducing recognition by exoproteases, i.e., aminopeptidase and carboxypeptidase. Although most commonly the *N*-terminus is acetylated and the *C*-terminus is amidated, many other modifications hinder exoprotease-mediated hydrolysis [8].

### 2.2. Backbone

Aiming to extend the protection to endoproteases, the alpha amino acid backbone can be modified [9]. These peptidomimetic molecules can be generated via modifications such as isosteric replacement of amide bonds, amide alkylation, and carbon skeleton extension, among others [10]. The bioisosteric replacement consists in exchanging the amide bond for groups with similar biological properties such as esters (depsipeptides), thioesters (thiodepsipeptides), or even triazole groups. Other examples of modifications that can be performed to a natural peptide backbone are α-methylation and *N*-methylation. While α-methylation of amino acids rigidifies the peptide structure and has an effect on the peptide dihedral angles, which is especially critical in helical peptides, *N*-methylation decreases hydrogen bond formation and increases lipophilicity, thereby enhancing the peptide capacity to cross biological barriers [11]. The backbone can also be modified using β or γ amino acid residues, which extend the carbon skeleton and expand conformation and folding possibilities [10].

### 2.3. Side Chains

An alternative to backbone modification for decreasing potentially both endo- and exoprotease recognition is the incorporation of amino acid residues with non-canonical side chains. There is a wide range of possible side chain modifications that have been found in nature or produced synthetically [12,13,14]. The most relevant family of non-canonical amino acids in targeting and cell-penetrating peptides is the family of D-enantiomeric amino acid residues. Peptides that are entirely composed of D-amino acids are generally known as “enantio” or “inverso” peptides. This kind of peptide has attracted increasing interest, owing to its high resistance to proteolytic degradation under physiological conditions [15]. Since the D-peptide shows different side chain orientation with respect to the parent L-peptide, target engagement may decrease if such binding depends on the 3D structure of the peptide. Combining enantio isomerization with reversing the peptide sequence results in the “retro-inverso” or “retro-enantio” isomer of the peptide (Figure 1, bottom left). The retro-enantio peptide displays a side chain topology similar to that of its native L-form with inverted amide bonds. For small peptides that do not depend heavily on tertiary structures, the retro-enantio isomer may have similar biological activity to the parent molecules, while presenting full resistance to proteolytic degradation [16]. Moreover, non-natural amino acids such as D-amino acids may decrease potential immunogenicity [17].

### 2.4. Cyclization

While modification of peptide side chains, backbone, and termini can provide outstanding resistance to proteolytic degradation, they often result into lower peptide affinity. Conversely, cyclization may increase not only hydrolytic stability but also binding to the target protein [18,19,20,21,22]. Cyclic peptides often show improved biological activity compared with their linear counterparts since the active conformation can be favored, thereby decreasing the entropic component in binding [23]. In addition, the constraint induced by cyclization decreases the probability of a good fit in the active site of endoproteases [5]. This shielding against enzymatic hydrolysis is highly efficient in short cyclic peptides and in longer sequences in which secondary structures, such as turns, helices, and sheets, are stabilized by the cyclization [20]. Amide bonds that are part of a hydrogen-bonding network, as in stapled peptides, are particularly poor enzymatic substrates [24]. Furthermore, most cyclizations are also designed to involve the terminal residues; therefore, they also protect the peptide from exoproteases [25]. Depending on the peptide structural context, several cyclization strategies can be applied: head-to-tail, side chain-to-head, side chain-to-tail and side chain-to-side chain [5]. A plethora of chemistries have been developed for cyclization, ranging from natural disulfide bridges or isopeptide bonds to metal-catalyzed ring-closing metathesis (RCM), as well as azide–alkyne and Diels–Alder cycloadditions [26,27]. Moreover, peptide conformations may be further constrained by multicyclization and the use of chemical scaffolds to mediate linkage [28].

### 2.5. Conjugation

An alternative way of enhancing peptide resistance to proteases is via conjugation to another molecule or nanoparticle. This potential protection mode is intrinsic to targeting and cell-penetrating peptides that transport cargoes. It seems intuitive that the conjugation of the peptide will provide steric hindrance and decrease proteolytic degradation. However, although many studies have demonstrated and increased half-life upon conjugation to large carriers such as polyethylene glycol, antibody constant fragment, or nanoparticles, few analyze the integrity or function of the peptide with time. The conjugation site in the peptide is crucial for maximizing activity and minimizing proteolytic degradation, not only because different regions of the peptide are protected but also because conjugation may affect peptide conformational equilibrium [29]. Some therapeutic peptides conjugated to particular molecular entities such as gold and lanthanide nanoparticles can be rendered fully resistant to proteases [30,31,32]. However, this is not generalizable to all peptides and cargoes. Achieving such high resistance to proteases is particularly difficult to envision in the case of targeting and cell-penetrating peptides since they need to be exposed and able to bind receptors or interact with the cell membrane. Therefore, in the last decade, some of the strategies summarized in this section have been applied to enhance the stability of these peptides. Although some examples have been reported for different enhancement strategies, such as backbone modification [33], in the next sections we focus on the two most commonly used strategies in this context: enantio/retro-enantio isomerization and cyclization (Table 1).

## 3. Enantio and Retro-Enantio Isomerizations Enhance Targeting Efficiency by Increasing Resistance against Proteolytic Degradation

### 3.1. Enantio/Retro-Enantio Cell Penetrating Peptides

**Figure 2:**
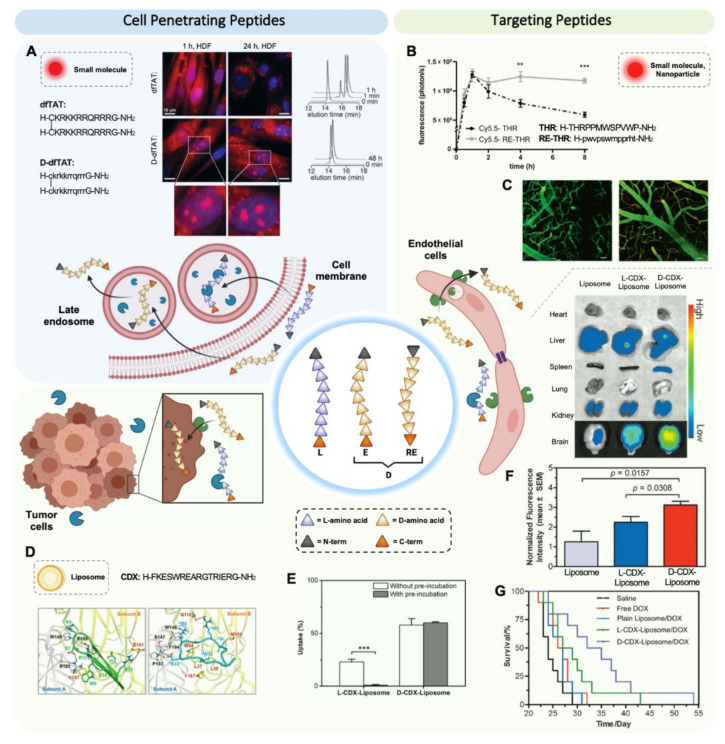
Enantio and retro-enantio isomerization enhance transport and targeting efficiency. (**A**) Cellular distribution of dfTAT and D-dfTAT immediately after delivery and after 24 h. Fluorescence images are overlays of the TMR (red) and Hoechst (blue) emissions (scale bars = 10 µm) (left). HPLC chromatograms of dfTAT and D-dfTAT upon treatment with trypsin (right), adapted from [37], Elsevier 2017. (**B**) In vivo fluorescence signal of the THR peptide in mice. Deviations represented as standard error mean. Unpaired t student test: ** *p* < 0.01, *** *p* < 0.001, adapted with permission from [39], John Wiley & Sons, 2015. (**C**) Intravital two-photon microscopy images of mice brains after injection of “naked” quantum dots (left) or QDs labeled with retro-enantio peptide (right) [39]. (**D**) Representation of the different binding modes of L-CDX (A, green) and D-CDX (B, aqua) with α7 nAChR. The subunit A of the receptor is shown in grey and subunit B in yellow. Residues involved in binding are represented with sticks, adapted with permission from [15], John Wiley & Sons, 2015. (**E**) Brain capillary endothelial cells uptake of CDX peptide-modified liposomes with and without preincubation in mouse serum. Quantitative cellular uptake by using flow cytometry [15]. (**F**) Ex vivo fluorescence quantification of brain and other organs of mice 8 h after injection of rhodamine B-labeled plain liposomes, L-CDX-liposomes, and D-CDX-liposomes (top). In vivo normalized fluorescence intensity of brain with biodistribution of rhodamine B-labeled D-CDX-liposomes, L-CDX-liposomes, and plain liposomes (bottom) [15]. (**G**) Survival plot of nude mice bearing intracranial U87 tumors. Mice that received four doses of D-CDX or L-CDX-modified liposomes encapsulating doxorubicin (DOX) survived significantly longer than the control groups that received saline [15].

D-amino acids have long been used to study the relevance of peptide 3D structure in peptide internalization. Prochiantz and collaborators, looking for the penetration mechanism of a 16 L-amino acid transcription factor fragment of Antennapedia, demonstrated the successful internalization of its D-version [54]. The fact that both L- and D-peptides could internalize in rat cortical-striatal E15 cells at 37 °C and 4 °C indicated that the internalization mechanism was not dependent on a chiral receptor. In this study, L-peptide was already observed to be more sensitive to proteolytic degradation at the higher temperature tested. Soon after, Wender, Futaki, and respective collaborators reported the potential of enantio isomers of arginine-rich cell-penetrating peptides (CPPs), which eventually became one of the most studied CPP families [34,55]. The efficient transport of these peptides is due to the electrostatic interactions between the positively charged arginine side chains and the negatively charged phospholipid membrane. In the pioneering study by Wender and coworkers, the D-versions (enantio and retro-enantio) of the arginine-rich membrane permeable HIV-1 Tat (49–57) were internalized in Jurkat cells more efficiently than the L-versions (parent and enantio) in the presence of 2% fetal bovine serum [34]. Under these conditions, the enantio version displayed a 3-fold higher cellular uptake than the parent peptide while in the absence of serum the uptake was equivalent, indicating that the chirality was not relevant for cellular uptake and that the higher internalization was due to the protease resistance of the D-version. Similar results were obtained by Futaki and collaborators, confirming the potential of the D-isomerization technique in CPPs [55]. Since then, this strategy has been applied to enhance the potential of many CPPs as drug delivery vectors. Remarkably, the protease resistance of the peptides is relevant not only to preserve the integrity of the peptide before reaching the cell membrane but also to enhance endosomal escape and intracellular fate [37]. Although several internalization mechanisms of CPPs have been reported, in endocytic pathways, hydrolytic resistance is clearly advantageous for resisting endosomal proteases and facilitating endosomal escape (Figure 2A).

CPPs stand as a versatile and powerful tool for enhancing the delivery of a wide variety of therapeutic cargoes. For instance, Liu et al. designed a chlorin gold nanorod derivatized with a D-rich arginine peptide for treating breast cancer by photothermal/photodynamic therapy [36]. CPPs have also been shown to enhance the cellular uptake of genetic material, including small interfering RNA (siRNA) [35] and RNA mimetics such as phosphorodiamidate morpholino oligomers (PMOs) [38]. D-CPP conjugates were completely stable in human serum for 24 h, while fragmentation of the natural L-CPP conjugates was observed within the first 2 h.

### 3.2. Enantio/Retro-Enantio Targeting Peptides

In contrast with CPPs, the 3D structure of targeting peptides is crucial in their interaction with receptors. Therefore, while CPPs exploit both the enantio and retro-enantio isomerizations, the vast majority of studies on targeting peptides focus on the retro-enantio approach, which aims to preserve side chain orientation. The retro-enantio approach has been used extensively to enhance the efficiency of peptides for the transport of therapeutics across the blood–brain barrier (BBB-shuttle peptides) and peptides targeting brain tumors [39]. This strong focus on brain delivery may be due to the high efficiency of targeting and transcytosis across brain endothelium required to reach brain targets in sufficient amounts [56].

One of the pioneering studies in the application of the retro-enantio approach to targeting peptides in this field, by Giralt and collaborators, aimed to enhance the efficiency of the 12-amino acid peptide THR (THRPPMWSPVWP), which binds the transferrin receptor (TfR1 or CD71) [57]. TfR1 is expressed in high levels on the brain microvasculature and enables transcytosis across the BBB. Although L-THR was able to facilitate the transport into the brain of gold nanoparticles to a limited extent [57], the unconjugated peptide was found to have a half-life of 30 min upon incubation in serum (Figure 2B) [39]. Three strategies were explored for enhancing the half-life of this peptide: *N-*methylation of most labile peptide bonds and enantio and retro-enantio isomerizations. Half-lives were 12 h for the *N-*methylated and above 24 h for the enantio and retro-enantio versions in human serum. The greater potential of the retro-enantio THR peptide (RE-THR) as a BBB shuttle was corroborated in mice. While the parent peptide and RE-THR conjugated to the fluorophore cyanine5.5 were shown to accumulate in the brain to a similar extent within the first hour, brain concentration of the first decreased by approximately 50% in 8 h, while the latter remained roughly constant in this time period. These results are consistent with the hypothesis that a small cargo such as cyanine5.5 conjugated at the *N-*terminus of the peptide could offer some protection from fast acting aminopeptidases but could not efficiently protect the sequence from endoproteases. Intravital two-photon microscopy showed that RE-THR was capable of driving quantum dots (QDs) into the brain parenchyma (Figure 2C). The free QDs remained strictly in the intravascular spaces of brain capillary, while RE-THR-conjugated QDs were detected outside brain vessels. Recently, Bukchin et al. corroborated the BBB shuttling efficiency of RE-THR peptide conjugated to amphiphilic polymeric nanoparticles [35].

The retro-enantio strategy has also been applied to Angiopep-2, the most advanced BBB shuttle in clinical trials [41]. Angiopep-2 is formed by 19 amino acids (TFFYGGSRGKRNNFKTEEY) and targets the low-density lipoprotein receptor-related protein 1 (LRP1). This peptide has been extensively used as a BBB shuttle and tumor-targeting peptide since LRP1 is expressed in high levels at the BBB and in several tumors, such as glioblastoma [58]. A retro-enantio isomer of L-Angiopep-2 was designed by Lu and collaborators to enhance its metabolic resistance. Micelles functionalized with retro-enantio Angiopep-2 displayed lower cellular uptake in a brain endothelial cell line than the L-parent peptide, suggesting a lower binding affinity to LRP-1. However, 50% of the parent peptide was degraded after a 30 min incubation in rat serum, while the retro-enantio analog displayed nearly no degradation after 8 h under the same conditions. Aligned with these results, the retro-enantio conjugate generated the highest brain distribution of micelles in mice 1 h and 4 h post injection. This indicates that, although the micelles might be partially shielding Angiopep-2 from degradation, the retro-enantio version increases its efficiency. Nonetheless, both retro-enantio and L-Angiopep-2 led to similar targeting of micelles in intracranial glioblastoma after 16 days post tumor implantation, which could be attributed to the BBB becoming compromised with the tumor progression in this mouse model, thereby decreasing the relevance of receptor-mediated transcytosis across brain endothelium. 

Ying et al. went one step further and showed that the retro-enantio approach could be applied to the L-A7R (ATWLPPR) peptide to achieve significantly higher tumor volume reduction in mice. L-A7R peptide displays a high affinity for vascular endothelial growth factor receptor 2 (VEGFR2) and neuropilin-1 (NRP-1), both of which are overexpressed on glioma and neovasculature [42]. The retro-enantio version (RE-A7R) showed similar affinity to the parent peptide but was barely degraded after a 4 h incubation in mouse serum, while L-A7R was practically undetectable within 2 h. When PEGylated liposomes were conjugated to each peptide, both conjugates could be efficiently internalized in vitro in glioblastoma (U87) and endothelial cell lines. Nevertheless, preincubation with mouse serum dramatically reduced L-A7R-liposome internalization, while RE-A7R-liposome internalization was not affected. A similar behavior was observed with the targeting ability in BTB/U87 tumor spheroids. These results suggest that the liposome conjugation would not provide effective protection to L-A7R against proteolytic cleavage, emphasizing the relevance of engineering the native peptide. In vivo, RE-A7R-liposome loaded with phenytoin showed around 70% higher accumulation in subcutaneous U87 tumors in mice than the L -isomer. Importantly this led to a more effective suppression of subcutaneous U87 tumor growth in mice than the L-conjugate. Furthermore, with the retro-enantio isomer, angiogenesis inhibition was also more pronounced (82% and 66%, respectively), and fewer vasculogenic mimicry channels were observed.

An even more compelling proof of the higher efficacy of retro-enantio peptides for brain tumor targeting was provided with a nanotherapy targeted with a retro-enantio version of VS peptide (VSWFSRHRYSPFAVS) [43]. VS targets α_6_β_1_ integrins, which are upregulated in glioma cells, and α_v_β_3_ integrins, which are overexpressed in cancer cells and neovasculature. In order to test the antitumor efficiency, VS-functionalized PEG-PLA micelles were loaded with doxorubicin and injected in nude mice bearing U87 tumors. Results confirm the potency of this approach in vivo because RE-VS doxorubicin-loaded micelles displayed higher tumor growth inhibitory effects than L-VS peptide-targeted control. Moreover, RE-VS-modified micelles resulted in the longest median survival in mice when compared with L-VS and the control groups: 22.5 for untargeted, 25.5 for L-VS targeted micelles, and 29.0 days for RE-VS targeted micelles.

One of the most complete studies of retro-enantio isomerization success in peptides capable of crossing the BBB and targeting brain tumors, also by Lu and coworkers, is that of L-CDX peptide (Figure 2D) [15]. This peptide targets the nicotine acetylcholine receptors (nAchRs) on the BBB and brain tumor cells. Receptor-mediated transcytosis of the retro-enantio CDX was higher than the parent peptide when conjugated to a PEGylated liposome. A 4 h preincubation in rat serum of the L-CDX-liposome conjugate almost nulled its cellular uptake, evidencing the liposome conjugation inefficiency for protection (Figure 2E). Moreover, the retro-enantio CDX version (dubbed D-CDX) displayed full resistance to rat liver lysosomal homogenate, while the L-version disappeared in 15 min. The authors reasoned that resistance to lysosomal degradation may alter intracellular transit of CDX conjugates, resulting in enhanced transcytosis. In addition, while most retro-enantio peptides tend to display lower affinities for their cognate receptor, retro-enantio CDX curiously enhanced its affinity for nAchR, 84.5 nM against 441.6 nM of the parent peptide. The authors argued that the higher affinity was due to a different binding mode, supported with molecular dynamics docking (Figure 2D). As a consequence of its higher stability and affinity, the retro-enantio CDX induced a 30% higher brain distribution compared with the L-analog (Figure 2F). This higher accumulation presumably resulted in a 20% increase in the median survival life of mice with glioblastoma compared with the L analog, and by 24% compared with the liposome-formulated doxorubicin (Figure 2G). Combined targeting efficiency of D-CDX and D-A7R has recently been confirmed in liposome drug delivery systems [59].

Most retro-enantio peptides have been obtained by isomerization of L-parent peptides. However, D-peptides can also be directly obtained using mirror-image phage display. An example of this in targeting peptides is the D-FNB peptide against the Fn14 receptor, which is overexpressed in many tumor cells [16]. To generate D-FNB, the D-enantiomeric form of the self-stabilized extracellular cysteine-rich domain of Fn14 membrane receptor was synthesized. An L-peptide was subsequently selected via phage display, and the D-enantiomer was synthesized. The in vivo antitumor efficacy of paclitaxel-loaded liposomes targeted with D-FNB significantly inhibited tumor growth in a subcutaneous xenograft model and drastically prolonged survival in a lung metastasis mouse model.

Although the retro-enantio approach may be capable of enhancing metabolic stability while maintaining biological activity, it is not a universally applicable strategy in targeting peptides since affinity for target receptors is often decreased. Mao et al. applied the enantio and retro-enantio approach to the AE peptide (FALGEA) to target the epidermal growth factor receptor (EGFR) and the mutation variant III (EGFRvIII) for glioma treatment [60]. The enantio and retro-enantio peptides were clearly more stable in serum incubation than L-AE. However, in terms of affinity, curiously, the enantio AE targeting capability was comparable with that of L-AE (K_D_: 2.0 μM and 5.0 μM, respectively), while retro-enantio AE showed a K_D_ = 211 μM. Although the enantio AE peptide was successfully used to target paclitaxel-loaded micelles, this study among others shows that despite its success, the retro-enantio strategy is not universally applicable, thus emphasizing the need for alternative ways to achieve metabolic stability, such as cyclization.

## 4. Cyclization May Enhance Targeting Efficiency Both by Providing High Protease Resistance and High Binding Affinity

Together with the use of D-amino acids, cyclization is the most common strategy for conferring protease resistance to targeting and cell-penetrating peptides. Although cyclization on its own does not guarantee the same degree of protection as enantio or retro-enantio isomerizations, it may enhance affinity for the target receptor if the binding conformation is stabilized [23]. As explained in Section 2.4, cyclization may decrease protease recognition provided enough rigidity is conferred to prevent a correct fit of the sequence in the enzyme active site.

### 4.1. Cyclic Cell Penetrating Peptides

**Figure 3:**
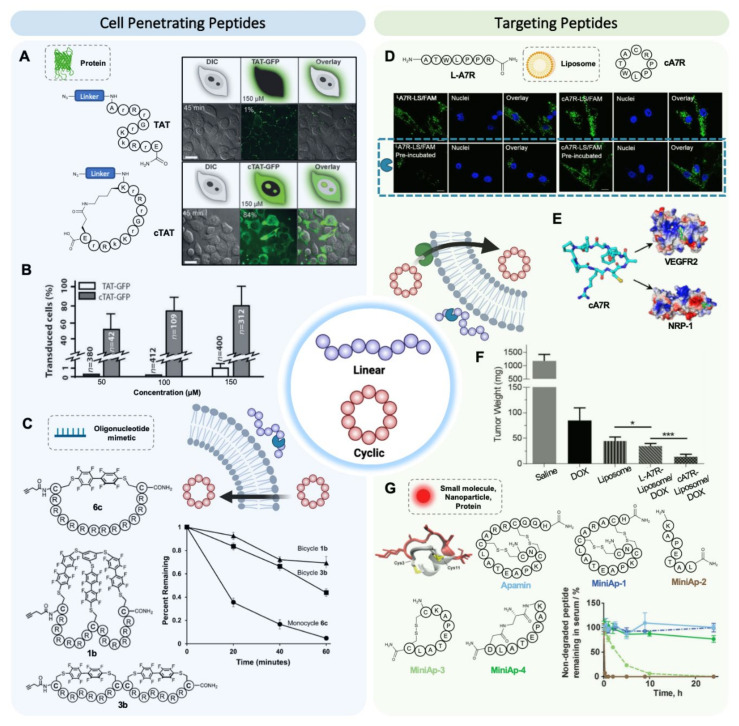
Cyclization of peptides enhances proteolytic stability and thus improves cell penetration or targeting. (**A**) Chemoselective conjugation via azide–alkyne cycloaddition of GFP to cTAT enhances transduction of living cells. Confocal microscopy images (scale bar = 15 µm) show efficient transduction of 84% of HeLa cells with the cTAT–GFP conjugate, compared with the 1% achieved by TAT–GFP, adapted with permission from [44], John Wiley & Sons, 2014. (**B**) Quantification of the percentage of transduced cells for 50, 100, and 150 µM of TAT–GFP and cTAT–GFP [44]. (**C**) Bicyclic peptides demonstrate enhanced proteolytic stability relative to monocyclic peptides after incubation with trypsin at 37 °C for up to 1 h, adapted with permission from [46], John Wiley & Sons, 2018. (**D**) Cyclic A7R-conjugated liposomes (LS) retain binding capacity to U87 cells after pre-incubation with 50% mouse serum for 4 h, while binding of L-A7R-LS is dramatically reduced, adapted with permission from [50], Elsevier, 2015. (**E**) 3D model of cA7R and its binding mode on VEGFR2 and NRP-1 receptors, as simulated by molecular docking [50]. (**F**) cA7R-conjugated LS loaded with doxorubicin (DOX) shows a very significant reduction in U87-derived xenograft weight in nude mice, confirming the positive influence of the cyclic structure on therapeutic efficacy [50]. (**G**) 3D representation of MiniAp-4. Proteolytic stability of the BBB shuttle apamin and its four derivatives MiniAp1–4 after incubation with 90% human serum at 37 °C for 24 h, adapted with permission from [52], John Wiley & Sons, 2015.

CPPs do not bind a receptor, but the cyclic structure has been shown to play a crucial role in the membrane partition efficiency and internalization mechanism [19,61]. This idea has recently been exploited by Cardoso, Hackenberger, and collaborators to enhance the activity of arginine-rich CPPs such as TAT. Cyclic versions of these peptides enhanced non-endocytic cellular uptake, improving internalization kinetics [44]. Cyclic TAT peptide entered living cells on average 15 min earlier than the linear form and showed a higher cell accumulation. This behavior was attributed to the higher separation between guanidinium groups in the cyclic conformation. The cyclization of this arginine-rich peptide not only improved its kinetic performance but also proved to be a suitable strategy for translocating large cargoes such as GFP (Figure 3A,B) and recombinant anti-GFP nanobodies in vitro [44,62]. Cyclic TAT experienced at least a 5-fold increase in the percentage of transduced HeLa cells with one of the nanobodies compared with the linear version. Along the same lines, Parang and collaborators applied the cyclization approach to the amphipathic CPPs (WH)_5_. The cyclic peptide increased the cellular uptake of two different cargoes, the cell-impermeable phosphopeptide GpYEEI and the anti-HIV drug FTC, by 4- and 2.6-fold, respectively, compared with the linear peptide, thus proving to be a more efficient molecular transporter [45].

Taking the cyclic design one step further, Pentelute and coworkers have recently developed bicyclic CPPs for delivering oligonucleotides into the cell nucleus. The peptide macrocyclization links the cysteine sidechains of unprotected peptides with perfluoroaromatic linkers in a stapled peptide mode. The monocyclic and bicyclic peptides improved conjugated phosphorodiamidate morpholino oligonucleotide (PMO) exon-skipping activity, both of them roughly 14-fold over the unconjugated PMO in HeLa cells. However, bicyclic peptides demonstrated over 10-fold greater stability than the monocyclic peptide in trypsin (Figure 3C), so the bicyclic versions are expected to have higher activity in vivo [46]. The higher shielding of the bicyclic analog is most likely due to the length of the sequence, the conformation of which is not stabilized enough in the monocyclic version.

### 4.2. Cyclic Targeting Peptides

Cyclization may also enhance the efficiency in targeting peptides. This enhancement is not only due to an increased resistance to proteases but also to a higher affinity for the target receptor, originating from the stabilization of the binding conformation. Although contribution to the targeting efficiency from each one of these phenomena is often not dissected, experiments across several studies illustrate the relevance of cyclization in targeting efficiency.

One of the first and most studied receptor-targeting peptides is RGD (Arg-Gly-Asp). This sequence presents a high affinity towards integrins, a large family of cell adhesion receptors that are widely used in cancer targeting due to their abundance on tumor cells and vasculature [63,64]. The RGD sequence was first identified in the fibrinogen protein [65], and since then, many variants have been developed. The most commonly used variant is a 5-residue cyclic peptide, RGDfK, with enhanced affinity and stability [66]. Liu et al. have recently generated dual-cyclic RGD-peptide derivatives with increased selectivity for targeting monomethyl auristatin E (MMAE) to tumor cells. These peptide–drug conjugates display 2-fold higher cytotoxicity than unmodified MMAE [47]. The RGD motif has also been incorporated into larger peptide structures such as knottins, whose highly constrained structure also confers high proteolytical stability. Cochran and collaborators obtained the engineered knottin EETI 2.5F, with high selectivity for integrin receptors, which provided outstanding imaging contrast for mouse cerebellar medulloblastoma [48]. This same peptide conjugated to gemcitabine proved to be a potent inhibitor of brain, breast, ovarian, and pancreatic cancer cell lines in vitro [67].

A well-studied example of the favorable conformation-locking mediated by cyclization includes the NGR sequence. This tripeptide targets CD13, a tumor marker associated with the myeloid linage. Molecular dynamic simulations indicate that the turn induced by cyclization populates the receptor-binding conformation, enhancing affinity [68]. To support this simulation, a cyclic version of this peptide with a disulfide bridge (CNGRC) and the linear version GNGRG were conjugated to tumor necrosis factor alfa (TNFα) and administrated to mice bearing B16F1 melanoma. The efficacy of the cyclic peptide was 10-fold that of the linear one. These results were attributed to the higher affinity and metabolic stability of the more rigid cyclic peptide, although the contribution of each mechanism was not dissected. More recently, Dreher and collaborators synthesized the cyclic peptide cKNGRE, in which the *N*-terminus was linked to the side chain of glutamate on resin forming a side chain-to-tail lactam bridge [49]. When conjugated to temperature-sensitive liposomes, the cyclic peptide resulted in a 3.5-fold higher affinity than the linear one. The fluorescently labelled cyclic analog also had a dramatically higher capacity to penetrate HT-1080 cells. Although the protease resistance of the peptides was not evaluated, the in vitro imaging tests were carried out in serum (30 min incubation). Therefore, the higher fluorescence signal may be partly attributed to the higher stability of cyclic peptides.

Cyclization has also been applied to longer peptides such as the heptapeptide A7R (ATWLPPR), which binds two glioma markers, VEGFR2 and NRP-1 (Figure 3E) [42]. Lu and coworkers showed that the cyclic version of L-A7R could enhance the anti-glioblastoma effect in vivo by conjugating it to doxorubicin-loaded liposomes [50]. The L-A7R peptide is fully degraded within 2 h upon incubation in mouse serum. Conversely, head-to-tail cyclization enhanced the lifetime of this peptide up to 12 h. In vitro, both linear and cyclic peptides improved liposome uptake in U87 glioma cells and different kinds of endothelial cells. In contrast, after incubation in serum, while the performance of the cyclic peptide conjugate was unaltered, the one of the linear peptide conjugates was clearly decreased (Figure 3D). When the liposomes were loaded with doxorubicin, the cA7R constructs exhibited higher anti-tumor and anti-angiogenic effects in nude mice U87 xenografts (Figure 3F).

Another clear example of the improved targeting capacity of a long cyclic peptide is embodied by the M2pep peptide (YEQDPWGVKWWY). M2pep targets M2 tumor-associated macrophages (TAM), which promote cancer proliferation by suppressing the immune response and stimulating angiogenesis and tumor growth, thus being a potential target for cancer therapies. [69]. Linear M2pep selectively internalizes in M2 macrophages but in no other leukocytes. Upon fusion with the proapoptotic KLA peptide, M2pep reduces the TAM population, prolonging median survival in a murine colon carcinoma model [70]. Nevertheless, low binding affinity and serum stability limit its efficacy as targeting moiety. In a follow-up study, two metabolically stable variants were synthetized: a C-acetylated version (AcM2pep(RY), which was fully degraded within 8 h, and a head-to-tail cyclized variant (M2pep(RY)) with a half-life beyond 24 h in serum [51]. The latter was able to promote significantly higher uptake of the fluorescent dye sulfoCy5 in M2-like TAMs, both in colon and mammary carcinoma models.

Cyclic peptides have also proven to be superior BBB shuttles that selectively enhance the delivery of cargoes into the brain [56,71,72]. A prominent example is that of apamin (CNCKAPETALCARRCQQH), a naturally occurring bicyclic peptide in bee venom that can circumvent the BBB and deliver several cargoes [71,73]. The main problem with the bicyclic natural peptide as a targeting moiety is its neurotoxicity and high immunogenicity. This is why several analogs (MiniAp-1 to 4) were synthesized mimicking the region in the peptide involved in BBB transport and not originating toxicity (Figure 3G) [52]. While the half-life of apamin in human serum was over 24 h, the half-life corresponding to the linear analog was as low as 5 min (Figure 3G). A monocyclic version with a disulfide bridge had an intermediate half-life of roughly 3 h. Remarkably, by simply replacing the disulfide by a lactam bridge, bridging the side chains of an *N*-terminal diaminopropionic acid and a *C*-terminal aspartic acid, the half-life increased beyond 24 h, close to that of apamin and a bicyclic analog. The lactam-bridged MiniAp-4 displayed higher BBB permeability than apamin and negligible toxicity and immunogenicity. MiniAp-4 is capable of enhancing the transport of GFP, imaging quantum dots, and gold nanoparticles in a cell-based model of the BBB. Furthermore, this cyclic BBB shuttle improved by 8-fold the accumulation of a fluorescent probe in mouse brain. Recently, other venom-inspired cyclic BBB shuttles such as miniCTX have been developed [74].

## 5. Conclusions and Outlook

Although many linear all-L peptides have been shown to provide efficient targeting, numerous studies prove that enhancing peptide resistance to proteolysis may further boost targeting and cell penetration. Susceptibility of peptide to degradation depends on the amino acid sequence, cargo, and conjugation site. Among the plethora of strategies developed to enhance the resistance of peptides to proteases, two of them have been applied to targeting peptides and CPPs in the vast majority of cases: enantio/retro-enantio isomerization and cyclization. Full enantio and retro-enantio isomerization intrinsically provides very high resistance to peptide degradation. Interestingly, this may alter intracellular trafficking of peptides, which is especially relevant in CPPs and in shuttle peptides that mediate transcytosis across the blood–brain barrier. Therefore, this isomerization approach has been extensively used in brain-targeted therapeutics, especially addressing brain tumors. Although many successful retro-enantio targeting peptides have been reported, these peptides generally display substantially lower binding affinity. By contrast, cyclization often enhances peptide affinity for its target if the adequate conformation is favored, although increase in protease-resistance may be more limited. By fine-tuning the cyclization strategy, peptides with high selectivity and protease resistance can be engineered.

Despite the numerous examples supporting the use of protease-resistant peptides for targeting and cell penetration, the contribution to protease shielding from sequence modification is often difficult to dissect from that provided by conjugation to the cargo. This is due to many studies lacking a clear comparison between the cargo conjugated to both the parent peptide and the “chemically enhanced” version. In the case of the cyclic peptides, there is an additional factor to be taken into account: the effect of cyclization on affinity. To this end, more data on the stability and the affinity of free and conjugated peptides should be provided. Additionally, as it has been recently noted [6], assays to assess peptide stability should be homogenized to enable comparison across studies.

Overall, in recent years numerous studies have proven that modifications increasing protease resistance can significantly enhance the efficiency of targeting and cell penetration peptides, both in cells and in vivo. A careful choice of controls and conditions in future studies will facilitate more accurate comparisons.

## Figures and Tables

**Table 1:** Representative enantio/retro-enantio and cyclic targeting and cell-penetrating peptides.

Name	Sequence	Modification	Application	Cargoes	Reference
d-Tat _49–57_ d-Tat_57-49_	rkkrrqrrr rrrqrrkkr	Enantio Retro-enantio	Cell internalization	Small molecules, nanoparticles, proteins, oligonucleotides	[34,35,36]
D-dfTAT	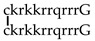	Enantio	Cell internalization	Small molecules	[37]
D-R_9_F_2_C	rrrrrrrrrffc	Enantio	Cell internalization	Oligonucleotides	[38]
THRre	pwvpswmpprht	Retro-enantio	BBB-shuttle	Small molecules, nanoparticles	[39,40]
^D^Angiopep	cyeetkfnnrkGrsGGyfft	Retro-enantio	Brain tumor targeting	Micelles	[41]
^D^A7R	rpplwta	Retro-enantio	Brain tumor targeting	Liposomes	[42]
^D^VS	svafpsyrhrsfwsv	Retro-enantio	Brain tumor targeting	Micelles	[43]
^D^CDX	GreirtGraerwsekf	Retro-enantio	BBB shuttle and brain tumor targeting	Liposomes	[15]
D-FNB	eGakhGltfsGG	Retro-enantio	Tumor targeting	Liposomes	[16]
cTAT	K(&)rRrGrKkRrE(&)	Cyclization	Cell internalization	Proteins	[44]
(WH)_5_	&WHWHWHWHWH&	Cyclization	Cell internalization	Peptides and small molecules	[45]
Arginine rich peptide (1b)	C(&)RRRRRRC(&)RRRRRRC(&)*	Cyclization	Cell internalization	Oligonucleotides	[46]
Cyclo(RGDfK)	&RGDfK&	Cyclization	Tumor targeting	Cytotoxic drug monomethyl auristatin E (MMAE)	[47]
EETI 2.5F	GC(&_1_)PRPRGDNPLTC(&_2_)SQDSDC(&_3_)LAGC(&_1_)VC(&_2_)GPNGFC(&_3_)G	Cyclization	Tumor targeting	Small molecules	[48]
cKNGRE	K(&)NGRE(&)	Cyclization	Tumor targeting	Proteins, liposomes	[49]
cA7R	&CATWLPPR&	Cyclization	Brain tumor targeting	Liposomes	[50]
Cyclic M2pep(RY)	C(&)GYEQDPWGVRYWYGC(&)kkk	Cyclization	Targeting of tumor-associated macrophages	Small molecules	[51]
MiniAp-4	(Dap)(&)KAPETALD(&)	Cyclization	BBB shuttle	Proteins, nanoparticles, small molecules	[52]

Cyclic peptide nomenclature was adapted from [53] * Trifunctional chemical scaffold.

## Data Availability

Not applicable.

## Abbreviations

BBB	Blood–brain barrier
CPP	Cell penetrating peptide
DOX	Doxorubicin
EGFR	Epidermal growth factor receptor
EGFRvIII	Epidermal growth factor receptor mutation variant III
FDA	Food and Drug Administration
FTC	2’,3’-dideoxy-5-fluoro-3’-thiacytidine (Emtricitabine)
GFP	Green Fluorescent Protein
HIV-1	Human immunodeficiency virus type I
HPLC	High-performance liquid chromatography
LS	Liposomes
MMAE	Monomethyl auristatin E
nAchRs	Nicotine acetylcholine receptors
NRP-1	Neuropilin-1 receptor
PEG	Polyethylene glycol
PMO	Phosphorodiamidate morpholino oligonucleotide
QD	Quantum dot
RCM	Ring-closing metathesis
RE	Retro-enantio
siRNA	Small interfering RNA
TAM	Tumor-associated macrophages
TfR1	Transferrin receptor
VEGFR2	Vascular endothelial growth factor receptor 2
